# Study of the Role of Titanium and Iron Cathodic Cages on Plasma-Nitrided AISI 430 Ferritic Stainless Steel

**DOI:** 10.3390/mi13101739

**Published:** 2022-10-14

**Authors:** Mirza Z. Babur, Aiyah S. Noori, Zafar Iqbal, Muhammad Shafiq, Muhammad Asghar, Abdulaziz H. Alghtani, Vineet Tirth, Ali Algahtani, Abid Zaman

**Affiliations:** 1 Department of Physics, Riphah International University, Islamabad 44000, Pakistan; 2 Medical Physics Department, Al-Mustaqbal University College, Hillah 51001, Iraq; 3 Department of Physics, Quaid-i-Azam University, Islamabad 45320, Pakistan; 4 Department of Mechanical Engineering, College of Engineering, Taif University, Taif 21944, Saudi Arabia; 5 Mechanical Engineering Department, College of Engineering, King Khalid University, Abha 61421, Saudi Arabia; 6 Research Center for Advanced Materials Science (RCAMS), King Khalid University Guraiger, Abha 61413, Saudi Arabia

**Keywords:** plasma nitriding, titanium cathodic cage, ferritic stainless steel, surface hardness

## Abstract

In contrast to austenitic and martensitic stainless steels, ferritic stainless steels have a lower hardness and wear resistance but exhibit excellent corrosion resistance. Due to this fact, their use in the aerospace, automobile, and house construction industries is restricted. Several methods have been utilized to enhance the tribological characteristics of ferritic stainless steels. In this work, titanium nitride coating has been carried out by using a cathodic cage of titanium material, and later on, the titanium cathodic cage is replaced by an AISI-304 cathodic cage in a CCPN chamber to form iron nitride coating on AISI-430 ferritic stainless steel coupons through a plasma nitriding process for 4 h at a fixed temperature of 400 °C. The microstructures and mechanical traits of all processed and control coupons were analyzed using scanning electron microscopy, X-ray diffraction, ball-on-disc wear tester, and microhardness tester techniques. The results showed that hardness increased up to 1489 HV with the titanium cage, which is much higher than the hardness of the base material (270 HV). The titanium cage-treated coupons have high layer thickness, smooth surface morphology, and a minimum crystallite size of 2.2 nm. The wear rate was reduced up to 50% over the base material after the titanium cage plasma treatment. The base coupon exhibited severe abrasive wear, whereas nitrided coupons exhibited dominant adhesive wear. In the iron nitride coatings, this effect is also important, owing to the more influential cleaning process in a glow discharge, and the better adhesion with enhanced interlayer thickness is attributed to the fact that the compliance of the interlayer minimizes shear stresses at the coating–substrate interface. The use of a graded interface improves adhesion compared with the case where no interlayer is used but a titanium interlayer of comparable thickness provides a significant increase in measured adhesion. For both titanium and iron nitride films, there is a reduction in wear volume which is a function of interlayer thickness; this will have a substantial effect on wear lifetime. Thus by careful control of the interlayer thickness and composition, it should be possible to improve coating performance in tribological applications.

## 1. Introduction

Stainless steel is distinguished from other forms of steel by the presence of chromium, nickel, and other alloying elements, which provide it with improved corrosion resistance. Ferritic stainless steel is a nonhardenable straight chromium class of stainless alloys with chromium levels ranging from 10% to 30% and less than 20% carbon content. Heat treatment does not significantly harden these steels, and cold rolling only mildly hardens them [1]. These alloys are used in car exhaust pipes, inner and outer beautification, kitchen tools, the automotive industry, and other aero-technological applications with low cost, easier availability, and high corrosion resistance [2]. Unfortunately, the lower hardness and poor wear resistance of such alloys limit their use as compared to austenitic and martensitic stainless steels. There are many techniques to increase the wear resistance of these ferritic stainless materials, such as plasma-nitro-carburizing (PNC) processes [3,4,5,6] and plasma nitriding (PN) processes. In contrast to the PN method, in which just nitrogen gas was used, in the PNC method, both nitrogen and carbon gases were utilized by injecting methane or acetylene during the treatment process. A small part of the published literature has been focused on the PN and PNC treatment of ferritic steels such as AISI 405, AISI 430, AISI 439, and AISI 441 [7,8]. Due to higher nitrogen absorption in the ferrite network than in the austenite phase, Oliveira et al. [9] and Tuckart et al. [10] found that the PN treatment of AISI 405 and AISI 439 ferritic steels at 500 °C generates a thick homogeneous coating layer with iron and chromium nitride phases. Gontijo et al. [11] revealed that the plasma nitriding of AISI 409L at a lower temperature (≤450 °C) results in the α_N_ phase with the supersaturated solid form of nitrogen in the ferritic phase [11]. Several methods have been utilized to improve tribological properties. These methods include physical vapor deposition [12,13,14], magnetron sputtering [12,13], an ion beam-assisted process [15], and chemical vapor deposition at higher temperatures [16]. Many researchers have examined the creation of nitride by different duplex surface treatments in recent years, such as chromizing, aluminizing [17,18], and titanizing [19] by the pack cementation technique, followed by plasma nitriding. However, there is little information about the production of niobium nitride films using this approach [20]. Furthermore, earlier research [21,22] revealed that the treatment sequence used in various duplex treatments might have a considerable impact on the coating’s microstructure and characteristics. In addition, slurry coatings [23,24,25], cathodic electro-deposition, and the anodic electro-deposition of metals and alloys, followed by annealing in open air [26,27,28] and pack cementation [29], have also been reported in the literature. Pack cementation is essentially an in situ, spontaneous CVD process [30]. CVD is an economical process used to produce an intermediate layer on various alloys in which deposition executes with different elements [31,32,33]. This process has many benefits, such as being easy to run, consistent, having effective surface protection from oxidation and corrosiveness, the high adhesiveness of the deposited layer to the specimen of variable size and geometry, and forming a smooth coating of customized thickness [33]. Unluckily, this process requires 6–10 h at high temperatures, limiting such coatings due to the undesired impact on the mechanical characteristics of the sample surface. Moreover, Pack cementation also has the disadvantage of oxidized and brittle covering due to the production of inter-metallic materials [34,35,36]. Coatings are created by heating a ferrous substrate surrounded by a combination of metal and halide salt particles in this approach. When the halide powder combines with the metal powder, evaporable metal compounds are formed, which dissolve when they come into touch with the ferrous substrate [37]. Naeem et al. [38] reported a comparison of surface hardness, wear, and corrosion behavior between the CCPN-, PVD-TiN-, and duplex-treated coupons. In this report, maximum surface hardness with better wear results has been obtained.

In this report, a study of titanium coatings during the plasma nitriding process by using a cathodic cage of titanium material (say, Ti CC) compared with austenitic stainless steel cathodic cage-based processed coupons (say, ASS CC) iron cage is performed. According to the author’s knowledge, there are no published results on the plasma nitriding of AISI-430F steels using an iron cage and a titanium cage.

## 2. Experimental Details

### 2.1. Materials

Samples of AISI 430F stainless steel with a thickness of 3 mm were cut into pieces with a surface area of 12 mm^2^. All coupons had their surfaces fine-polished with various grades of silicon carbide sheets. For 30 min, the polished coupons were immersed in acetone in an ultrasonic bath.

Table 1 shows the chemical composition of the AISI 430F ferritic steel utilized in this study.

### 2.2. CCPN System

**Figure 1:**
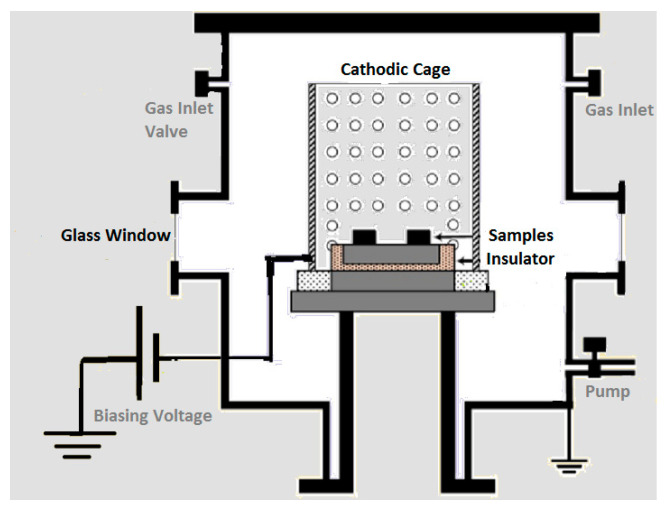
Schematic of CCPN treatment system.

**Figure 2:**
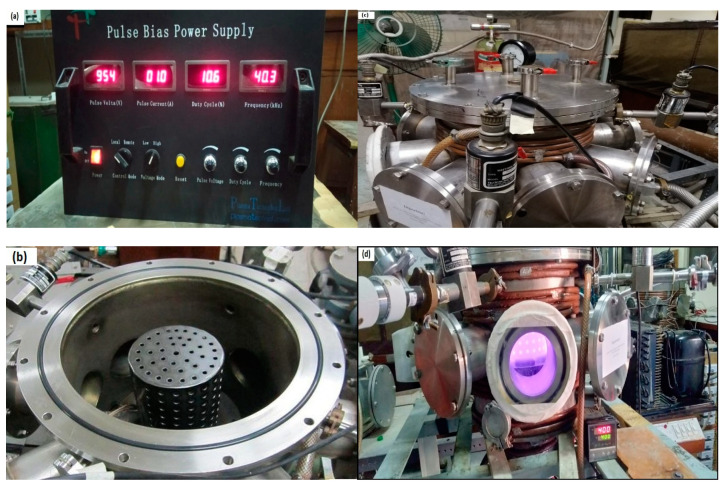
A pictorial view of (**a**) pulsed bias power supply, (**b**) CCPN system without a lid, (**c**,**d**) CCPN system during plasma treatment.

CCPN equipment’s complete description can be found in earlier reports [39]. Figure 1 shows a schematic representation of the whole CCPN system. In the CCPN system, there is a cylindrical chamber composed of AISI-304 stainless steel with an inner diameter and altitude of ~310 mm and 335 mm, respectively. The chamber’s top circumference is engraved to seat the rubber gas kit to achieve an airtight system, with a stainless steel lid with the same diameter being utilized to cover the chamber tightly by using nut-bolts as shown in Figure 2b. A heater was mounted outside the chamber but in contact with it thermally, whereas chilled water was circulated around the chamber through copper pipes to avoid thermal leakages and insulation damage. The grounded chamber’s walls were electrically connected with an anode terminal and cathode terminal that were connected with an active screen, which is why this is also termed a cathodic cage. This was utilized to develop a smooth nitrogen film as well as to minimize the overheating of the coupons and act as a counter electrode. In this study, two types of cathodic cages have been utilized for the surface treatment in the CCPN chamber one by one; the first was AISI-304 stainless steel, and the other was composed of titanium alloy. Both have dimensions of approximately 80 mm and 130 mm in height and diameter along with equidistant holes of ~8mm on the eccentric surface and 6mm on top. The space between the coupons and the top of the cathodic cage was kept at 4.5 cm.

### 2.3. Deposition of Coatings and Plasma Parameters

In the CCPN chamber, the base coupons were first put on a ceramic platform. Prior to the start of the plasma processing, the chamber was kept empty through a rotary vane pump up to ~1 Pa, which can be visible on a Pirani gauge, whereas a conventional gauge was used to show the working gas pressure. To eliminate oxides and other contaminants on the surface, the coupons were first treated with a plasma of argon gas at 350 °C for 20 min. After that, a temperature of 400 °C was achieved by heater and retained for 4 h during CCPN treatment by admixing a N_2_/H_2_ = 3/2 ratio. The flow rates of the argon, nitrogen, and hydrogen gases were set at 2 sccm, 28 sccm, and 20 sccm, respectively, by maintaining the 150 Pa of the chamber’s pressure, opting for the previously optimized conditions [39,40]. The specimens were insulated from the cathodic cage during the plasma treatment and kept on floating potential. The plasma electrodes were created using a 40.3 kHz power source with a pulsed voltage of 954V and a pulsed current of 1.0 A along with 10.6% of a pulsed duty cycle [41] as shown in Figure 2a.

The cathodic cage of iron of AISI304 stainless steel was used initially in the plasma treatment process, and then the same treatment process was repeated by using a cathodic cage composed of titanium on the other fresh coupons to make a comparison between the titanium and iron coatings, as shown in Figure 2b. Nitrogen was used as a source gas for the generation of the nitriding species, along with hydrogen and argon, in the treatment process. The nitrogen ions created during plasma exposure filled in the basic materials’ interstitial locations. The mechanical characteristics of the plasma-nitrided coupon were then compared to the base and TiN-coated coupons.

## 3. Structural and Mechanical Characteristics

A Vickers micro-hardness tester (HV-30, RAYTECH, China) has been employed to investigate the changes in the surface hardness of the base and plasma-processed coupons. A diamond indenter, in the form of a right pyramid with a square base and with a specified angle between the opposite faces at the vertex, is forced into the surface of a test piece, followed by the measurement of the diagonal length with a pyramid shape formed by indentation with an indent angle of 136° of the planes by the exertion of loads (10–1000 N) for 15 s. According to the relation given below:

HV = 1.854 × F/d^2^

where, F = Normal Load (N) and d^2^ = Indentation Area (mm^2^). In case the area of indent is not so clear, then the means of a large number of readings is reported to achieve precision. In this article, the average data have been compiled to calculate the eleven hardness measurements for each sample. An X-ray diffractometer (JDX-3532, JEOL, Tokyo, Japan) with a Cu-Kα radiation source (1.5406 Å) was used to find the surface structural study of the treated and untreated specimens. The surface texture and the severity of wear grooves on the base and plasma-processed coupons were studied using a scanning electron microscope (SEM) (JSM-5910, JEOL, Tokyo, Japan) before and after wear track testing. The wear qualities of a steel ball with a diameter of 6 mm were studied using a pin-on-disc technique with a load of 10 N and a sliding speed of 8.87 cm/s for ~560 s. These experiments were carried out three times and then averaged. Steel balls had bulk and shear moduli of 140 and 80 GPa, respectively, and an elastic modulus of 190–210 GPa. The ASTM G99 standard was used to analyze the wear volume and rate [42].

## 4. Results and Discussion

### 4.1. Surface and Cross-Sectional Morphology

**Figure 3:**
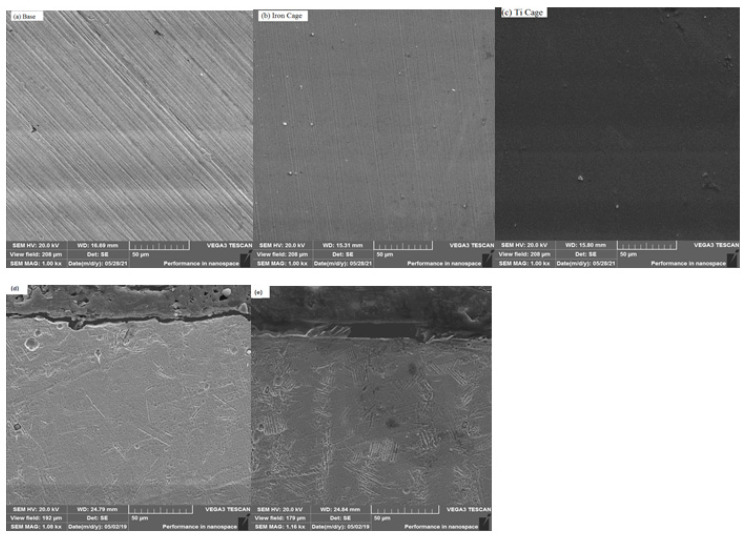
Scanning Electron Micrographs of (**a**) base (**b**) ASS CC (**c**) Ti CC whereas (**d**) ASS CC and (**e**) Ti CC cross-sectional images.

The surface morphology, obtained from Scanning Electron Microscopy analysis, is revealed in Figure 3a–c. Some trenches on the surface of the base coupon were observed due to mechanical polishing, as described in Figure 3a. In the nitrided samples (Figure 3b) with the ASS CC, scratches are entirely covered with a deposited coating layer of the uniform nitride layer, and the few white particles have a random distribution. The white particles shown in the high-resolution picture are caused by the aggregation of iron nitride particles, a process that has also been observed in steel alloys [43]. This aggregation of nitride precipitates is greater in the ASS CC specimen than in the Ti CC sample, presumably due to the increased nitrogen content. The cross-sectional morphology is also presented in Figure 3d,e. It reveals that the developed nitride layers with thicknesses of ~3.37 μm and ~5.81 μm are due to the ASS CC and Ti CC, respectively, due to the sputtered Ti n-depth diffusion of nitrogen. This is confirmed by the hardness profile, which depicts the enhanced layer thickness of Titanium nitride. Because the hardness profiles and nitrogen concentration have a linear connection and a comparable depth [44], we may assume that in-depth nitrogen diffusion occurs in the current investigation exactly like in-depth hardness improvement. There were some random minor holes that appeared in Figure 3d which can be attributed to the coupon’s position or deposition parameters, also observed by Panjan et al. [45]. Furthermore, Figure 3e shows the thick and uniform titanium nitride layer attributes such as the higher hardness and the reduced wear rate with better adhesion, which depicts superior results to previously conducted experiments by Kao et al. [46], who adopted the CFUBMS titanium-coating technique.

### 4.2. Microhardness with Indentational Toughness Analysis

**Figure 4:**
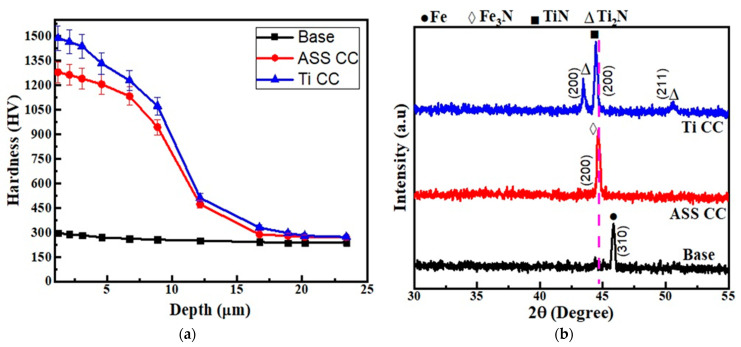
(**a**) Micro-hardness patterns of base and nitrided coupons and (**b**) XRD profile.

The Vickers microhardness of the base AISI 430F steel coupon is demonstrated in Figure 4a, which shows a change in the surface hardness with respect to the indentation depth. It shows a 270 HV microhardness value of the untreated coupon, whereas the plasma-nitrided coupons with the iron and titanium cages have microhardness values recorded as 1280 HV and 1489 HV, respectively, as reported in Figure 4a. This depicts the Titanium cage-based coating has the highest hardness value compared to the untreated as well as the iron cage-based coatings, which are approximately 5.5 and 4.7 times harder than the base coupon. This precipitous improvement in surface hardness is attributed to the formation of hard titanium nitride precipitates [47]. It is worth noting that, despite the thin layer existing in the titanium cage-based coated specimens, the microhardness values increased when compared to the plasma-nitrided specimens. The existence of iron nitrides, as well as carbon- and nitrogen-saturated ferrite phases, is responsible for this [48], whereas Krishna et al. [49] and Ananddan et al. [50] conducted the surface alloyed titanium and WC+Ni+NiCr treatments on AISI304 stainless steel for 6h each and the hardness of ~718 HV and ~1320 HV was achieved, respectively. On the other hand, in this work, 1489HV of hardness was obtained in AISI430 stainless steel with titanium coating by the CCPN technique, which attributes to enhanced results in 4 h treatment time.

### 4.3. Phase Analysis

Glancing incidence XRD spectra are shown in Figure 4b for the untreated and the CCPN-treated coupons by utilizing the iron cage in one experiment and the titanium cage in another experiment. The presence of just the ferrite phase with peak (310) has been recorded for untreated AISI 430F steel samples. The coupons displayed (200) phase peaks of iron nitride, which were observed after CCPN surface treatment with the iron cage. This phase is a supersaturated solid solution of nitrogen in the ferritic phase. Gontijo et al. [11] made a similar observation with AISI 409L ferritic steel plasma-nitrided at 350 °C and 400 °C. The Fe_3_N phase is found in the BCC lattice due to the minimal nitrogen absorption [51]. On the other hand, titanium nitride peaks (200) and (211) with slightly lower 2θ values were recorded in an XRD pattern of the coupons plasma-treated at 400 °C with the titanium cage, and the titanium cage was employed as a cathode during this procedure. Furthermore, the existence of ferrite peaks in the XRD pattern in addition to those corresponding to TiN indicates that the modified layers generated by the Ti cage at that temperature may be finer than those obtained with the iron cage-treated specimens. This agrees with Kale et al. [47] where titanium was diffused in the coupon’s surface without the formation of the compound at the interlayer but these compounds were developed at higher temperatures of ~955 °C [48].

### 4.4. Crystallite Size and Micro-Strain

**Figure 5:**
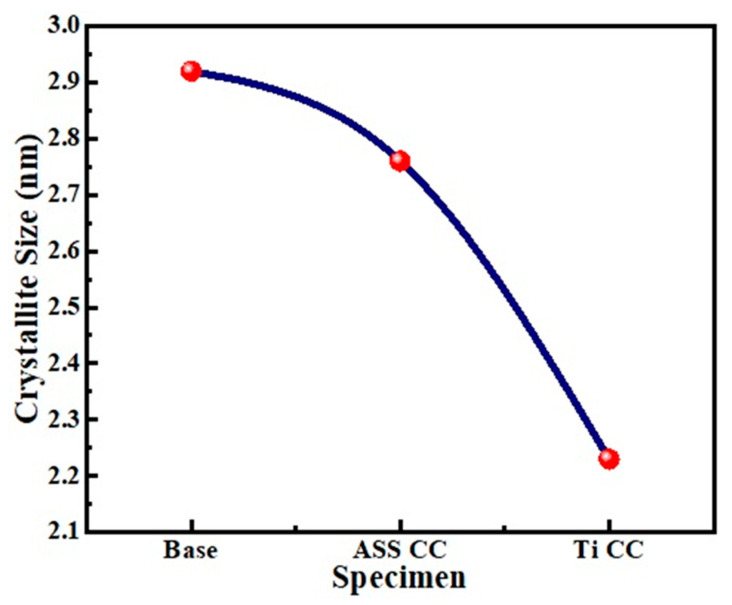
Change in crystallite size with specimen processed type.

To investigate the crystalline size, the Scherrer Equation was applied, i.e.,

*L* = *Kλ*/*β* ∙ cosθ (1)

where, *L* is the nano-crystallite size (*L*) by the XRD radiation of the wavelength *λ* (nm) from measuring the full width at half of the maximum of the peaks (*β*) in radian located at any 2θ in the pattern, and K is the Scherrer constant [52]. The crystalline size may be determined from the XRD peaks (Figure 4b) of the base at (310), the ASS CC at (200), and the Ti CC at (200), a single peak for each coupon using the above-mentioned equation. The variation in crystalline size with the ASS CC- and Ti CC-treated specimens is depicted in Figure 5, which depicts that the crystallite size is small when the Ti CC is used as compared to Iron CC. As demonstrated in Figure 4b, the broadness of the ASS CC- and TI CC-based processed coupons were observed, which is due to the diffusion of nitrogen and titanium atoms into the steel matrix causing lattice distortion. The minimal crystalline size is confirmed by the Ti CC-treated coupons, which showed maximum hardness. The Hall Petch equation (H = H_o_ + k′d^−1/2^) reveals an increase in surface hardness with a decrease in crystalline size [53,54]. The diffusion of nitrogen ions into interstitial sites, lattice distortions (strain), and flaws are all contributing to this tendency, and this is caused by a higher ion flow in the base material. The micro stresses may also be created in the lattice structure and are used to broaden the peaks and improve the hardness [55].

### 4.5. Wear Volume and Wear Rate

**Figure 6:**
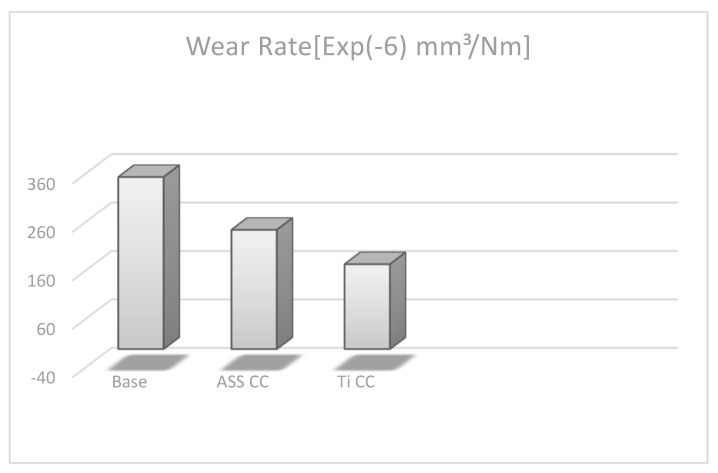
Wear rate diagram of plasma treated and untreated coupons.

**Figure 7:**
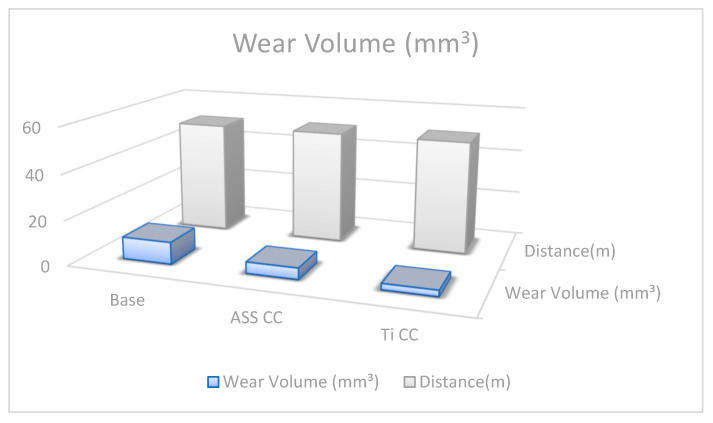
Wear volume pattern of plasma treated and untreated samples.

The samples underwent real analysis, and from the results, the quantitative wear rate and wear volume plots are given in Figure 6 and Figure 7, which depict the wear mechanism of the ASS CC- and Ti CC-treated coupons. The following relation was used to estimate the wear volume of the base and plasma-processed coupons [56]:

ΔV = 2πR × [r^2^ × arcsin(w/2R) − (w/4){4r^2^ − w^2^}^1/2^]

In this equation, R, w, and r stand for wear track radius, wear track width, and radius of the counter face ball, respectively. All are measured in millimeter units. Figure 7 illustrates that the base coupon shows the max volume track (1.49 × 10^−8^ mm^3^) due to the deep and wide wear track indicating severe wear. Furthermore, the wear volume of the Ti CC- and ASS CC-treated coupons has been recorded as 3.1 × 10^−8^ mm^3^ and 5.09 × 10^−8^ mm^3^, which shows improved surface hardness versus the untreated coupon. This phenomenon is also confirmed by the microhardness plot (Figure 4a), which indicates that the Ti CC-treated coupon has maximum hardness due to a thicker coating layer on the substrate material than that of the ASS CC and base coupons. However, the ASS CC-treated coupon showed significant improvement in tribological properties but less than the Ti CC-treated coupon due to the narrow and trivial wear track during the wear test. In addition, the following relationship is used to calculate the wear rate [56]:

η = ΔV/FS

In this equation, F(N), ΔV, and S(m) stand for the normal load, wear volume, and total sliding distance, respectively. According to this relation, the wear rate (η) is directly proportional to the wear volume values, and from the above discussions, it is found that the wear volume of the base coupon has a maximum value (3.55 × 10^−4^ mm^3^/Nm) shown in Figure 6. On the other hand, the Ti CC-treated coupon has recorded the minimum wear rate value (1.75 × 10^−4^ mm^3^/Nm), which agrees well with the relation of the wear rate by indicating an excellent wear response among the tested specimens. It depicts that the best wear results in the shape of the wear rate and the wear volume of 2.65 mm^3^ have been achieved by Ti CC by CCPN processing. It demonstrates that the wear rate obeys Archard’s equation, which states that wear rate and surface hardness have an inverse relationship, as stated by Farahnaz et al. [57].

In the same manner, the wear volume of the Ti CC-treated coupons is recorded as 3.65 times less than the base with the minimum wear volume, which is due to the hard layer of TiN, as shown in Figure 7, which shows a strong adhesivity of the titanium nitride interlayer with a hardness of ~1500 HV.

## 5. Conclusions

The effect of the active-screen plasma-nitrided surface treatment on ferritic stainless steel by using an austenitic stainless steel cathodic cage (ASS CC) and a titanium cathodic cage (Ti CC) has been evaluated and the following conclusions are proposed:

(1) The thickness of the nitriding layer was increased more by utilizing the titanium cathodic cage (Ti CC) than the austenitic stainless steel cathodic cage (ASS CC) by ASPN treatment for an equal treatment time.

(2) The improved surface hardness, minimum crystallite size, and better film quality of the ASPN-treated samples with the Ti CC were recorded.

(3) The process is very cost-effective as it uses a single setup with a short time and low temperature to obtain maximum hardness with better wear behavior than the earlier report [38].

(4) The plasma treatment improved the wear resistance of the coupons with improved coating thickness with the use of the titanium cathodic cage in the CCPN chamber.

(5) The crystalline size is reduced for the Ti CC compared to the base and ASS CC coupons and hence obtaining the enhanced surface properties.

(6) Wear properties have been recorded to be enhanced remarkably, which is the confirmation of Archard’s equation.

(7) The wear volume of the Ti CC-treated coupons has been recorded as 0.5 times the wear volume of the base coupons with a minimum wear rate of ~175 × 10^−6^ mm^3^/Nm.

## Figures and Tables

**Table 1:** Chemical composition of AISI430 ferritic steel sample.

Elements	C	Cr	Si	Mn	P	S	Fe
Composition (Wt%)	0.10~0.12	16.2~17.1	0.37~0.5	0.92~1.17	0.02	0.32	Balance

## Data Availability

Generated data should be publicly available and cited in accordance with journal guidelines.
